# Polymorphisms in the uncoupling protein 3 gene and their associations with feed efficiency in chickens

**DOI:** 10.5713/ajas.18.0217

**Published:** 2018-05-31

**Authors:** Sihua Jin, Lei Yang, Tingting He, Xinfeng Fan, Yiqiu Wang, Kai Ge, Zhaoyu Geng

**Affiliations:** 1 College of Animal Science and Technology, Anhui Agricultural University, Hefei 230036, China

**Keywords:** Uncoupling Protein 3, Polymorphism, Feed Efficiency, Association, Chicken

## Abstract

**Objective:**

The uncoupling protein 3 (UCP3) is a member of the mitochondrial anion carrier superfamily and has crucial effects on growth and feed efficiency in many species. Therefore, the objective of the present study was to examine the association of polymorphisms in the *UCP3* gene with feed efficiency in meat-type chickens.

**Methods:**

Six single nucleotide polymorphisms (SNPs) of the *UCP3* gene were chosen to be genotyped using matrix-assisted laser desorption-ionization time-of-flight mass spectrometry in meat-type chicken populations with 724 birds in total. Body weight at 49 (BW49) and 70 days of age (BW70) and feed intake (FI) in the interval were collected, then body weight gain (BWG) and feed conversion ratio (FCR) were calculated individually.

**Results:**

One SNP with a low minor allele frequency (<1%) was removed by quality control and data filtering. The results showed that rs13997809 of *UCP3* was significantly associated with BWG and FCR (p<0.05), and that rs13997811 had significant effects on BW70 and BWG (p<0.05). Rs13997812 of *UCP3* was strongly associated with BW70, FI, and FCR (p<0.05). Furthermore, individuals with AA genotype of rs13997809 had significantly higher BWG and lower FCR (p<0.05) than those with AT genotype. The GG individuals showed strongly higher BW70 and BWG than AA birds in rs13997811 (p<0.05). Birds with the TT genotype of rs13997812 had significantly greater BW70 and lower FCR compared with the CT birds (p<0.05). In addition, the TAC haplotype based on rs13997809, rs13997811, and rs13997812 showed significant effects on BW70, FI, and FCR (p<0.05).

**Conclusion:**

Our results therefore demonstrate important roles for *UCP3* polymorphisms in growth and feed efficiency that might be used in meat-type chicken breeding programs.

## INTRODUCTION

In modern poultry production, feed represents approximately 60% of the total raising costs especially in developing countries. In addition, low feed efficiency chickens produce too much excreta which may result in environment pollution [1]. Therefore, it is imperative for researchers and breeders to select superior feed efficient birds in poultry breeding programs. With the rapid development of whole genome sequencing technologies, molecular and physiological characterization of key candidate genes affecting growth and feed efficiency traits can be used as molecular markers for the genetic improvement of broiler chickens [2,3].

The uncoupling proteins (UCPs) located in the mitochondrial inner membrane are members of anion carrier protein superfamily and regarded as crucial regulators of energy homeostasis [4]. Briefly, UCPs are composed of five isoforms in mammals, including UCP1, UCP2, UCP3, UCP4, and UCP5 [5]. Among these genes, uncoupling protein 3 (*UCP3*) is mainly expressed in skeletal muscle and plays a vital role in regulating energy metabolism at transcriptional and posttranslational levels [6]. In chickens, the UCP3 gene is physically located on chicken chromosome 1 and consists of 6 exons and 5 introns spanning 2.9 kp in the vicinity of quantitative trait loci (QTL) influencing growth and feed conversion ratio (FCR) trait (https://www.animalgenome.org/cgi-bin/QTLdb/GG/index) [7,8].

It is well documented that UCP3 protein decreases metabolic efficiency via uncoupling substrate oxidation in mitochondrial from ATP synthesis by mitochondrial respiration chain, thus dissipating mitochondrial proton gradient by mediating inducible mitochondrial proton leak to regulate energy metabolism [9]. In poultry, the *UCP3* gene is mainly expressed in skeletal muscle and plays crucial roles in energy metabolism, limiting reactive oxygen production, fatty acid metabolism, and body weight (BW) [5,10,11]. In humans, a missense polymorphism in codon 55 of the *UCP3* gene is significantly associated with obesity susceptibility [12]. It was reported that genetic mutation of g.C4902T in the *UCP3* gene is significantly correlated with partial body measurement traits including withers height and chest depth in Chinese Qinchuan cattle [13]. Another study showed that relative expression of *UCP3* in the low feed efficiency group is significant higher than that in the high feed efficiency group in beef cattle [14]. In addition, previous research demonstrated that avian UCP mRNA expression in breast muscle of the low feed efficiency chickens is higher than that of high feed efficiency birds [15].

Taken together, numerous studies regarding the relationships between *UCP3* gene polymorphisms and growth, feed efficiency and energy metabolism disorders have been performed in mammals and humans [16,17]. Until now, there have been few studies on associations of the *UCP3* gene with growth and feed efficiency traits in chickens. The objective of the present study was to investigate the association of polymorphisms in the *UCP3* gene with BW, body weight gain (BWG), feed intake (FI), and FCR in meat-type chickens, which may be helpful as molecular markers in future breeding programs to improve growth and feed efficiency of meat-type chickens.

## MATERIALS AND METHODS

### Ethics statement

All animal experiments were performed in accordance with the Regulations for the Administration of Affairs Concerning Experimental Animals (Ministry of Science and Technology, China, revised in June 2004) and approved by the Institutional Animal Care and Use Committee of Anhui Agricultural University (permit number: IACUC-20101020). All experimental protocols were carried out according to relevant regulations and recommendations by this committee. All efforts were made to minimize suffering in our birds.

### Birds and data collection

Seven hundred and ninety-six males from the 22nd generation of two yellow meat-type chicken strains were chosen as experimental populations in the present study. These included 335 chickens from N202 strain and 461 birds from N301 strain with both strains having complete pedigree information of three generations (G_20_ to G_22_). The two strains were selected based on the appearance, growth and carcass traits within every generation and chosen as valuable genetic sources for a local chicken breeding program. All chickens were derived from a single hatch, wing-banded at day of hatch and raised indoors. All birds were given the same experimental diet (Table 1) provided by Wens Nanfang Poultry Breeding Co. Ltd., Guangdong, China and had the same feeding protocols. Feed and water were freely available during the whole experiment period. At 42 days of age, all birds were transferred into the individual cages with the size of 25×40×45 cm and special troughs used for recording individual FI. Body weight at 49 (BW49) and 70 (BW70) days of age and FI in the interval were measured individually. The BWG and FCR were calculated according to FI and BW in the interval from 49 to 70 days of age. At the end of the trail, a total of 724 birds (439 chicks from N301 and 285 chicks from N202 strain) were used for association analysis of *UCP3* polymorphisms with BW49, BW70, FI, BWG, and FCR. However, we removed 72 chickens owing to their incomplete genomic or phenotypic information.

### Single nucleotide polymorphisms selection, genotyping and quality control

The single nucleotide polymorphisms (SNPs) in the *UCP3* gene were obtained from UCSC Genome Browser database (http://genome.ucsc.edu/cgi-bin/hgGateway). The analysis of all the SNPs was performed by the BLAST program at the NCBI database and DNAMAN 7.0 (Lynnon Biosoft, Quebec, Canada) to guarantee all the SNP sequences were non-homologous associated with other genome sequences. Finally, six SNPs in the *UCP3* gene were selected for further analysis.

All blood samples were collected from the wing vein by standard venipuncture and stored in acid citrate dextrose anticoagulant at −20°C before DNA isolation. Genomic DNA was extracted from whole blood samples using NRBC Blood DNA Kit (Omega, Norcross, GA, USA) in accordance with the manufacturer’s procedures. DNA quality was tested by 1.5% agarose gel electrophoresis and concentration was quantified using the NanoDrop 2000 spectrophotometer (ThermoFisher Scientific, Waltham, MA, USA). The final DNA concentrations were 2 to 10 ng/μL and stored at −20°C for further analyses. Two PCR primers and extension primer were designed using software Assay Design 3.1 (Sequenom, San Diego, CA, USA) for each SNP, as listed in Table 2.

Genotyping of 724 chickens was carried out by matrix-assisted laser desorption-ionization time-of-flight mass spectrometry on the Mass ARRAY iPLEX Platform (Sequenom, USA). Single nucleotide polymorphisms with a genotype call rate <90% and minor allele frequency <1% were discarded across all birds.

### Statistical analysis

The linkage disequilibrium (LD) between several SNPs in one gene was determined by the Haploview program [18]. Lewontin D′ statistic >0.8 and squared correlation coefficient r^2^>1/3 means sufficiently strong LD [19]. The haplotypes for SNPs in strong LD were inferred using Simwalk2 software (http://watson.hgen.pitt.edu/docs/simwalk2.html). The frequency of SNPs genotypes and Hardy-Weinberg equilibrium were analyzed by the FREQ procedure and chi-square fitness test of SAS version 9.4 (SAS Institute Inc., Cary, NC, USA). The SNPs that deviated from the Hardy-Weinberg equilibrium were removed for further analysis. The association between SNPs or haplotypes and growth, feed efficiency traits were analyzed using the generalized linear mixed model procedure of SAS 9.4 with the following model:

$${\text{Y}}_{\text{ijk}}=\mu +{\text{L}}_{\text{i}}+{\text{G}}_{\text{j}}+{\text{P}}_{\text{k}}+{\text{e}}_{\text{ijk}}$$

Where Y_ijk_ is the observed value of the traits, μ is the overall population mean, L_i_ is the fixed effect of line, G_j_ is the fixed effect of SNP genotype or haplotype, P_k_ is the random effect of family, and e_ijk_ is the random error.

## RESULTS AND DISCUSSION

Previous studies had demonstrated that the UCP3 was a member of the mitochondrial anion carrier protein families that play pivotal roles in energy metabolism and lipid metabolism [16,17]. Thus, many researchers had widely examined the possible function of the *UCP3* gene in energy homeostasis and its association with growth and feed efficiency in many vertebrates [20,21]. However, there was little published research on associations of *UCP3* polymorphisms with growth and feed efficiency traits in chickens. In the present study, six SNPs of the *UCP3* gene were chosen and screened in yellow meat-type chicken strains. One SNP with a very low minor allele frequency was removed by quality control and data filtering. The remaining five SNPs were further tested as polymorphic with genotype call rate >90% and minor allele frequency >1% (Table 3).

In the current study, association analysis revealed that three SNPs in the *UCP3* gene were significantly associated with growth and feed efficiency traits in chickens as listed in Table 4. Rs13997809 of the *UCP3* gene was significantly associated with BWG and FCR (p<0.05), and that rs13997811 was strongly associated with BW at 70 days of age (BW70) and BWG (p< 0.05). SNP rs13997812 had significant effects on BW70, FI, and FCR (p<0.05). Furthermore, LD analysis indicated a high linkage block among rs13997809, rs13997811, and rs13997812 of the *UCP3* gene (Supplementary Figure S1) and contributed to 99.81% of total haplotypes scored. They were H1 (AAC: 48.24%), H2 (AAT:5.04%), H3 (AGC:8.63%), H4 (AGT:7.23%), H5 (TGC:2.04%), H6 (TGT:3.56%), and H7 (TAC:25.07%), as given in Table 5. Association analysis also showed that the TAC haplotype was significantly associated with BW70, FI, and FCR (p<0.05, Table 5). The results indicated that the *UCP3* gene plays an important role in determining growth and feed efficiency traits in our chicken populations. Previous research had demonstrated that an A/G polymorphism in intron 3 of the *UCP3* gene is significantly associated with average daily gain, partial efficiency growth, and FCR in beef cattle [21]. It was reported that the *UCP3* gene has pivotal roles in uncoupling thermogenesis in beige adipocytes and might be a crucial mediator of thermogenesis in cold-tolerant pigs [22].

Previous studies demonstrated that the *UCP3* gene had crucial effects on energy expenditure and homeostasis, and their polymorphisms were significantly associated with fat metabolism, obesity and diabetes in humans and mice [16,23]. Therefore, it was regarded as an important candidate gene affecting growth and feed efficiency traits via regulating energy balance and lipid metabolism. In this study, individuals with the AA genotype of rs13997809 had larger BWG than those of the AT genotype, whereas FCR of the AA genotype was significant lower than those of the AT genotype (p<0.05, Table 6). Chickens with the GG genotype of rs13997811 showed strongly higher BW70 and BWG than those of the AA genotype (p<0.05, Table 6). For rs13997812, the TT individuals had significantly higher BW70 and lower FCR compared with the CT birds (p<0.05, Table 6). It was reported that polymorphisms in the *UCP3* gene were significantly associated with total cholesterol levels and genetic mutation in intron 1 may be correlated with relative expression levels of the *UCP3* gene in dogs [24]. In humans, recent studies reported that polymorphism of C55T in the *UCP3* gene might be associated with obesity [16]. Another study showed that *UCP3*/*Bgl*Ipolymorphism was significantly associated with growth traits, and genotype AB was superior over AA or BB genotypes in Nanyang cattle [25]. Therefore, the present study provided important reference for the effects of *UCP3* in chicken growth and feed efficiency.

In summary, three SNPs and the TAC haplotype of the *UCP3* gene were significantly associated with some growth and feed efficiency traits, which might be used as potential genetic markers in yellow meat-type chicken breeding programs. Further studies are necessary to properly investigate associations of *UCP3* polymorphisms with feed efficiency traits in large populations with different chicken strains. Additionally, it is necessary to further investigate the molecular mechanisms of identified SNPs or haplotypes of the *UCP3* gene contributing to growth and feed efficiency of chickens.

## Supplementary Data



## Figures and Tables

**Table 1:** Analyzed nutritional components of the experimental meat-type chicken rations

Nutrient1)	49–70 d
Metabolizable energy (kcal/kg)	2,837
Crude protein (%)	20.36
Crude fiber (%)	4.15
Calcium (%)	0.85
Available phosphorus (%)	0.63
Methionine (%)	0.39
Lysine (%)	0.64

1) Feed and analyzed nutrient composition were provide by Wens Nanfang Poultry Breeding Co., Ltd., Guangdong, China.

**Table 2:** Primer sequences used in genotyping for the *UCP3* gene

SNP	Primer	Sequence (5′ to 3′)
rs13997809	Forward	ACGTTGGATGGGGACCTTATAGAGACCTAC
	Reverse	ACGTTGGATGACCTCAATAAGCTCTCACAC
	Extension	GTCACACAGGAGCAGC
rs13997810	Forward	ACGTTGGATGAATCCCCACCCCTTCACGG
	Reverse	ACGTTGGATGTGACACAACCCCAAAGTCCC
	Extension	CGCCGCCCTCCCCTCAGAG
rs13997811	Forward	ACGTTGGATGTGTCCCCACTCGGTGGCAT
	Reverse	ACGTTGGATGAAGTGAGGCTGCAGGTAAAG
	Extension	CTATTGAGCTCCCACAGCGC
rs13997812	Forward	ACGTTGGATGAAGTGAGGCTGCAGGTAAAG
	Reverse	ACGTTGGATGTGCCCCAGCTGTCCCCACT
	Extension	CCCACTCGGTGGCAT
rs1058384613	Forward	ACGTTGGATGTTGAATGTGAGAGGAGAAGG
	Reverse	ACGTTGGATGTCCTCTGTCAATGCCATCTC
	Extension	AGCCCCACAATGACA
rs315264270	Forward	ACGTTGGATGGAGGGGAATGGTTTTTGTGG
	Reverse	ACGTTGGATGGTCTGAGAGCTACAATCGTG
	Extension	GTTGTGGTTTTGTTTTGTTTTGTT

*UCP3*, uncoupling protein 3; SNP, single nucleotide polymorphisms.

**Table 3:** Genotypic and allelic frequency of SNPs in *UCP3* and Hardy-Weinberg equilibrium test

SNP	Location	No. of chickens	Genotypic frequency			Allelic frequency		p-value for HWE
	
			AA1)	AB	BB	A	B	
rs13997809	Promoter	686	0.30	0.52	0.18	0.56	0.44	0.6485
rs13997811	Promoter	703	0.30	0.46	0.24	0.53	0.47	0.3949
rs13997812	Promoter	682	0.26	0.54	0.20	0.53	0.47	0.3536
rs13997810	Promoter	708	0.44	0.42	0.24	0.65	0.35	0.3363
rs1058384613	Exon	654	0.30	0.44	0.26	0.52	0.48	0.1149
rs315264270	5′-UTR	704	0.98	0.02	0	0.99	0.01	0.9655

SNP, single nucleotide polymorphisms; *UCP3*, uncoupling protein 3; HWE, Hardy-Weinberg Equilibrium test.

1) AA means the dominant allele.

**Table 4:** Association analysis of *UCP3* polymorphisms and haplotypes with growth and feed efficiency traits in meat-type chickens

Growth and feed efficiency traits1)	p-value for significant test					

	rs13997809	rs13997810	rs13997811	rs13997812	rs1058384613	TAC haplotype
BW49	0.3957	0.4394	0.1439	0.0674	0.2466	0.2163
BW70	0.0832	0.9822	0.0161*	0.0074**	0.3049	0.0271*
BWG	0.0328*	0.2359	0.0024**	0.0684	0.9079	0.0676
FI	0.0687	0.7795	0.1124	0.0187*	0.7149	0.0347*
FCR	0.0124*	0.1354	0.0510	0.0206*	0.5746	0.0149*

*UCP3*, uncoupling protein 3.

1) BW49, body weight at 49 days of age; BW70, body weight at 70 days of age; BWG, body weight gain from 49 to 70 days of age; FI, feed intake from 49 to 70 days of age; FCR, feed conversion ratio from 49 to 70 days of age.

* p<0.05,

** p<0.01.

**Table 5:** Haplotypes based on SNPs of rs13997809, rs13997811, and rs13997812 in the *UCP3* gene

No.	Haplotype	Observations	Frequency (%)
H1	AAC	713	48.24
H2	AAT	75	5.04
H3	AGC	127	8.63
H4	AGT	107	7.23
H5	TGC	30	2.04
H6	TGT	52	3.56
H7	TAC	371	25.07

SNPs, single nucleotide polymorphisms; *UCP3*, uncoupling protein 3.

**Table 6:** Least squares means±SE for growth and feed efficiency traits among genotypes of three SNPs in the *UCP3* gene in meat-type chickens

SNP	Genotype	Growth and feed efficiency traits1)				

		BW49 (g)	BW70 (g)	BWG (g)	FI (g)	FCR
rs13997809	AA(206)	1,101.02±4.32	1,712.55±4.40	611.35±3.53^a^	1,770.45±7.17	2.89±0.02^a^
	AT(356)	1,115.26±3.21	1,715.36±3.62	600.12±2.90^b^	1,770.15±3.89	2.95±0.01^b^
	TT(124)	1,116.20±2.35	1,720.21±4.58	604.21±3.78^ab^	1,774.15±6.47	2.92±0.02^ab^
rs13997811	GG(208)	1,110.93±4.13	1,720.37±4.65^A^	610.23±3.41^a^	1,786.46±5.75	2.93±0.04
	GA(325)	1,118.34±2.38	1,713.27±4.31^A^	595.84±2.74^b^	1,785.31±6.35	2.99±0.02
	AA(170)	1,108.54±3.40	1,702.41±4.47^B^	594.97±3.59^b^	1,779.26±6.96	2.99±0.03
rs13997812	CC(180)	1,108.45±4.19	1,716.15±3.57^ab^	608.54±3.64	1,775.99±6.63^a^	2.92±0.02^AB^
	CT(366)	1,112.43±3.83	1,708.69±4.63^a^	600.54±2.89	1,770.49±4.75^ab^	2.94±0.01^A^
	TT(136)	1,113.29±4.25	1,722.65±3.89^b^	609.03±3.75	1,763.68±7.62^b^	2.89±0.03^B^

SE, standard error; SNPs, single nucleotide polymorphisms; *UCP3*, uncoupling protein 3.

1) BW49, body weight at 49 days of age; BW70, body weight at 70 days of age; BWG, body weight gain from 49 to 70 days of age; FI, feed intake from 49 to 90 days of age; FCR, feed conversion ratio in the interval.

a–c Among genotypes within each SNP for each trait, means within a row with no common superscript differ (p<0.05).

A–C Among genotypes within each SNP for each trait, means within a row with no common superscript differ (p<0.01).
