# Supplementary material for: Polymorphisms in the uncoupling protein 3 gene and their associations with feed efficiency in chickens
Source: Asian-Australas J Anim Sci. 2018 May 31;31(9):1401–6. doi: 10.5713/ajas.18.0217 (PMC6127571; doi:10.5713/ajas.18.0217)
Supplement: Supplementary file 1 [file ajas-31-9-1401-supplementary.pdf]

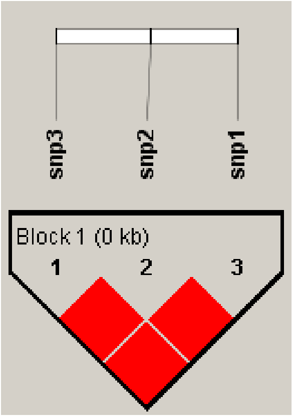

Supplementary Figure S1. The linkage status of three SNPs in the *UCP3* gene. SNP1, SNP2, SNP3 represents rs13997809, rs13997811, rs13997812 of the *UCP3* gene, respectively. The color of block reveals the LD status of SNPs; red color indicates high linkages among three SNPs.
